# CD44v8-10 is a marker for malignant traits and a potential driver of bone metastasis in a subpopulation of prostate cancer cells

**DOI:** 10.20892/j.issn.2095-3941.2020.0495

**Published:** 2021-08-15

**Authors:** Rosaria A. Fontanella, Silvia Sideri, Chiara Di Stefano, Angiolina Catizone, Silvia Di Agostino, Daniela F. Angelini, Gisella Guerrera, Luca Battistini, Giulia Battafarano, Andrea Del Fattore, Antonio Francesco Campese, Fabrizio Padula, Paola De Cesaris, Antonio Filippini, Anna Riccioli

**Affiliations:** 1 Department of Anatomy, Histology, Forensic Medicine and Orthopaedics, Unit of Histology and Medical Embryology, Sapienza University, Rome 00161, Italy; 2 Department of Health Sciences School of Medicine – “Magna Graecia” University of Catanzaro, Catanzaro 88100, Italy; 3 IRCCS Fondazione Santa Lucia, Rome 00143, Italy; 4 Bone Physiopathology Research Unit, Genetics and Rare Diseases Research Division, Bambino Gesù Children’s Hospital, IRCCS, Rome 00146, Italy; 5 Department of Molecular Medicine, Sapienza University, Rome 00161, Italy; 6 Department of Life, Health and Environmental Sciences, University of L’Aquila, L’Aquila 67100, Italy

**Keywords:** Metastasis, epithelial phenotype, EMT, MET, IL-6, TAZ

## Abstract

**Objective:**

Bone metastasis is a clinically important outcome of prostate carcinoma (PC). We focused on the phenotypic and functional characterization of a particularly aggressive phenotype within the androgen-independent bone metastasis-derived PC3 cell line. These cells, originated from the spontaneous conversion of a CD44-negative subpopulation, stably express the CD44v8-10 isoform (CD44v8-10^pos^) and display stem cell-like features and a marked invasive phenotype *in vitro* that is lost upon CD44v8-10 silencing.

**Methods:**

Flow cytometry, enzyme-linked immunoassay, immunofluorescence, and Western blot were used for phenotypic and immunologic characterization. Real-time quantitative polymerase chain reaction and functional assays were used to assess osteomimicry.

**Results:**

Analysis of epithelial–mesenchymal transition markers showed that CD44v8-10^pos^ PC3 cells surprisingly display epithelial phenotype and can undergo osteomimicry, acquiring bone cell phenotypic and behavioral traits. Use of specific siRNA evidenced the ability of CD44v8-10 variant to confer osteomimetic features, hence the potential to form bone-specific metastasis. Moreover, the ability of tumors to activate immunosuppressive mechanisms which counteract effective immune responses is a sign of the aggressiveness of a tumor. Here we report that CD44v8-10^pos^ cells express programmed death ligand 1, a negative regulator of anticancer immunity, and secrete exceptionally high amounts of interleukin-6, favoring osteoclastogenesis and immunosuppression in bone microenvironment. Notably, we identified a novel pathway activated by CD44v8-10, involving tafazzin (TAZ) and likely the Wnt/TAZ axis, known to play a role in upregulating osteomimetic genes.

**Conclusions:**

CD44v8-10 could represent a marker of a more aggressive bone metastatic PC population exerting a driver role in osteomimicry in bone. A novel link between TAZ and CD44v8-10 is also shown.

## Introduction

Prostate carcinoma (PC) is the most commonly diagnosed male malignancy and a leading cause of cancer death. About 5% of patients with PC have distant metastasis at the diagnosis^1^. PC commonly metastasizes to the bone and prostate and breast cancers are responsible for up to 70% of skeletal metastasis. These lesions cause clinically relevant skeletal complications resulting in pathologic fractures, spinal cord compression and debilitating bone pain. Bone metastases and relative complications are particularly problematic for patients with PC because, in these patients, the clinical course is relatively long-lasting and, after diagnosis, patients with PC survive 1 to 5 years^2^. Given the great impact of bone metastases on life quality of PC patients, we focused on the phenotypic and functional characterization of a previously identified subpopulation of bone metastasis-derived highly aggressive androgen-independent PC cells. These cells, only recently detected as a minor component of the bulk population of the PC3 cell line, once cultured in isolation were found to represent a promising model to get insight into the traits of PC metastatic potential^3^. This deceptively small population displays a CD44^neg^ phenotype but, once isolated and cultured *in vitro*, rapidly converts to a very aggressive phenotype, stably expressing *de novo* the CD44v8-10 isoform of CD44 (CD44v8-10^pos^). CD44v8-10^pos^ PC3 cells form colonies of holoclonal-type morphology, which is one of the stem cell characteristics previously demonstrated in PC3 cells^4^ and display a more invasive phenotype *in vitro* that is lost upon silencing of the CD44 variant^3^. CD44 is a single pass transmembrane glycoprotein involved in cell-cell interaction, cell-matrix adhesion and cell signaling. Its standard isoform (CD44s) is composed of 10 exons, but CD44 can undergo alternative splicing determining the expression of splicing variants involved in tumor progression and metastatic processes; CD44 variant (CD44v) isoforms derive from inclusion of additional exons, in different combinations, in the extracellular domain of the protein^5^. Interestingly, in a range of cancers, CD44v isoforms are expressed mainly in advanced stages^6^ and are associated with stem^7^ and metastatic^8,9^ features. On the contrary, increasing evidence is recently emerging of a controversial and isoform-specific role of CD44 in cancer. In particular, Zhang et al.^10^ have shown that CD44s and CD44v isoforms correlate with different cancer cell states and phenotypes: CD44s is closely associated with breast cancer stem cells (CSC) features and aggressiveness, whereas CD44v is negatively associated with these phenotypes. This theory is sustained by the fact that the isoform switch from CD44v to CD44s is functionally essential for cells to undergo epithelial–mesenchymal transition (EMT)^11–13^. Several studies have indicated a connection between CSCs and EMT^14,15^. EMT refers to a process by which epithelial tumor cells transform into mesenchymal cells in order to detach from the primary tumor, *via* loss of intercellular adhesion, gaining migratory and invasive abilities. At the same time, the reverse process, called mesenchymal–epithelial transition (MET), is necessary for cancer cell to redifferentiate once they colonize distant sites^16^. Evidence of MET in clinical specimens is the finding that the histology of metastatic tumors exhibits the epithelial phenotype rather than the mesenchymal-like phenotype, suggesting that the reversion of EMT occurs once the cells settle at the metastatic sites^17^. When metastatic cells from the prostate reach the bone, they can acquire phenotypic aspects of bone cells, a process known as osteomimicry^18^. Bone metastases are classified as osteolytic, osteoblastic, or mixed lesions^19^. Osteolytic lesions are characterized by excessive bone resorption around tumor sites and appear as focal areas of osteoporosis. On the contrary, osteoblastic lesions are associated with increased bone formation and appear sclerotic, resulting from increased osteoblasts differentiation and activity. Bone lesions resulting from PC are primarily osteoblastic, but are also associated with increased bone resorption^20^.

How cancer cells of epithelial origin adopt osteomimetic and osteotropic features and what are the clinical implications are unsolved questions, but it is known that tumor cells destroy the physiological bone remodeling impairing the normal osteoclast and osteoblast activity through the secretion of several cytokines and growth factors^21^. Thus, in osteolytic metastasis osteoclast activity is stimulated by cancer cells that release various cytokines, like interleukin (IL)-1 and IL-6, whereas in osteosclerotic bone metastasis, other cytokines and chemokines produced by cancer cells induce stimulation of osteoblasts^22^. Notably, several studies highlighted a key role of tumor cell-produced cytokines, such as IL-6, also in the pro-tumorigenic immunosuppressive network of bone microenvironment^23^.

Based on these findings, we sought to determine whether the CD44v8-10-expressing PC3 subpopulation with stem-like features and highly invasive ability display EMT/MET markers and osteomimetic phenotype and what is the role of the specific CD44v8-10 isoform. The focus of our aim was to characterize CD44v8-10 as a marker of more stem-like and highly metastatic PC cells for diagnosis or possibly as a target for therapeutic treatment. Interestingly, the association of a specific CD44 variant with metastatic progression or treatment resistance might make it a good candidate for selective cancer targeting, since CD44 variant isoforms are not as abundantly expressed in the normal tissue as the standard CD44^24^. Specifically, CD44v8-10 has been described in human colon cancer^25^ and in rat pancreatic and mammary cancer cells^26^ and is up-regulated in primary and metastatic tumors^9^ but rarely expressed in normal tissue. Moreover, CD44v8-10 is a specific CSC marker of head and neck^27^ and gastric cancers^28^. Recently, CD44v8-10 mRNA contained in serum exosomes was proposed as a diagnostic marker for docetaxel resistance in PC patients^29^.

Engagement of CD44, either alone or in combination with other surface receptors, activates a variety of intracellular signals, such as PI3K/AKT, MAPK, and survivin pathways^24,30^, thus regulating a number of oncogenic responses. Interestingly, CD44 has also been described as a regulator of the Wnt pathway, known to be involved in bone differentiation and metastasis^31^ and possible mediator of CD44v8-10-induced aggressiveness traits in our model.

In this study, we show that PC3 cells, which highly express CD44v8-10, display a frankly epithelial phenotype, high levels of intracellular basal reactive oxygen species (ROS), required for aggressive phenotype in PC; importantly, our data show that CD44v8-10 is not just a marker but a key player in the osteomimetic features of this cell population, enabling it to form bone-specific metastasis. Moreover, here we also report data on CD44v8-10-downstream signaling pathways underpinning the osteomimetic and metastatic features of CD44v8-10^pos^ cells; according to these data, the novel finding shows that this CD44 variant is involved in the tafazzin (TAZ)-related pathway, known to regulate the bone development. TAZ (WWTR1) is the effector of the HIPPO pathway and is frequently linked to cancer progression and metastasis in various tumors^32–35^. High levels of TAZ are observed in 20% of human tumors, where it induces the expression of several secretory factors as connective tissue growth factor and cysteine-rich angiogenic inducer 61 (CYR61), important in stromal interactions and metastatic niche formation, and ankyrin repeat domain 1 (ANKRD1)^36^.

Our results suggest that CD44v8-10 should be considered in the long-sought effort to devise targeted therapies against PC bone metastasis.

## Materials and methods

### Cell lines and primary human cells

Human peripheral blood mononuclear cells (PBMCs) were from healthy donors. The experiments with PBMC are in collaboration with Prof. Salvatore Minisola (Sapienza University, Rome, Italy) in accordance with the rules set by our institutional review board (Policlinico Umberto I – Ethical Committee, Rif. 3040/16.01.2014 protocol, approval No. 73/14), including the donors’ signature of informed consent. Concerning human osteoblasts from bone fragments, the source of human bone cells was exclusively waste byproducts from our orthopedic surgery, which were otherwise to be discarded. Our experiments therefore required no harvesting from patients; hence, no institutional approval was required.

PC3 (ATTC n. CRL-1435 Lot n. 61777391) cells were obtained from the American Type Culture Collection (ATCC, Manassas, VA, USA) and routinely checked for mycoplasma. Details of the isolation of the 2 subpopulations CD44v8-10^pos^ and CD44v8-10^neg^ cells by 3 sorting experiments of parental PC3 cells were previously described^3^. Cells were cultured in RPMI-1640 (Sigma, Saint Louis, MO, USA) supplemented with 2 mM L-glutamine (Sigma), 200 U/mL penicillin–streptomycin (Sigma), sodium pyruvate 1 mM (Sigma), HEPES 10 mM (Sigma), and 10% fetal bovine serum (FBS) (Life Technologies–Gibco, Eugene, OR, USA). They were maintained at 37 °C in a humidified 5% CO_2_ incubator.

### Flow cytometry

Cells were detached with 0.25% trypsin/EDTA (Sigma), washed with phosphate-buffered saline (PBS) 0.1% bovine serum albumin (BSA) and incubated with anti-human CD44v8-10 primary antibody (clone: RV3; Cosmo Bio Co. Ltd, Tokyo, Japan), PE-mouse anti-human EpCAM (clone: EBA-1; BD Biosciences, San Jose, CA, USA), APC-mouse anti-human E-cadherin (clone: 67A4; BioLegend, San Diego, CA, USA), mouse monoclonal anti-human Vimentin (Santa Cruz Biotechnology, Santa Cruz, CA, USA), mouse anti-human Citokeratin 18 (ThermoFisher Scientific, San Francisco, CA, USA), mouse anti-human pan-cytokeratins (clone: AE1/AE3; Dako, Glostrup, Denmark), BV421-mouse anti-human α2 Integrin (clone: 12F1; BD Biosciences), mouse monoclonal anti-human β1-integrin (clone: TS2/16, Santa Cruz Biotechnology), PE-mouse anti-human programmed death ligand 1 (PD-L1) (clone: 29E.2A3; BioLegend). All the primary antibodies were incubated in PBS 0.1% BSA for 30 min at 4 °C. APC-labeled goat anti-rat IgG (H + L) secondary antibody (Invitrogen, Carlsbad, CA, USA) was used for CD44v8-10, vimentin, CK18, pan-cytokeratins, and β1-integrin antibodies, and fluorescein isothiocyanate (FITC)-labeled goat anti-mouse IgG (H + L) secondary antibody (Invitrogen) was used for vimentin, CK18, pan-cytokeratins, and β1-integrin antibodies. Both secondary antibodies were diluted 1:800 in PBS BSA 0.1% for 30 min at 4 °C in the dark. Sytox Blue Dead Cell Stain (Life Technologies) was added to exclude dead cells. Cells were assayed using CyAn ADP flow cytometer (Beckman Coulter, Brea, CA, USA), and data were analyzed using FCS5 Express software (De Novo Software, Pasadena, CA, USA).

### RNA isolation, real-time quantitative polymerase chain reaction, and siRNA

Total RNA was extracted using TRIzol reagent (Invitrogen) according to the manufacturer’s instructions. cDNA was generated by High-Capacity RNA-to-cDNA TM Kit (Applied Biosystems–ThermoFisher, Waltham, MA, USA). PCR analyses were carried out using specific oligonucleotides for genes listed in **Supplementary Table S1**. Real-time quantitative polymerase chain reaction (RT-qPCR) was carried out on the StepOne Plus system and Q5 from Applied Biosystems using KAPA SYBR Green Fast assay mix (Merck, Darmstadt, Germany). The 2^−ΔΔCT^ method for relative quantization of gene expression was used to determine mRNA expression levels. *Beta-actin* gene expression was used as endogenous controls to standardize mRNA expression. *P*-values were calculated with 2-tailed Student’s *t*-test from at least 3 experiments. Statistically significant results are indicated by a *P*-value < 0.05.

SiRNAs targeting CD44v8-10 or the relative control siRNA (scramble) were transfected with RNAiMAX (Invitrogen) for 5 h according to the manufacturer’s protocol. After 72 h, the level of CD44v8-10 was evaluated by cytometry analysis, and if the CD44v8-10 silencing was > 70% (**Supplementary Figure S1**), RNA was extracted, reverse transcription was performed, and cDNA was used for qPCR. The siRNA sequences were previously described^37^ and are synthesized by Bio-FabResearch (Rome, Italy).

### ROS detection

Cells were detached with 0.25% trypsin/EDTA (Sigma), washed with PBS 0.1% BSA, and incubated with 200 μM H_2_O_2_ at 37 °C for 30 min. After 2 washes in PBS 0.1% BSA, the cells have been incubated with the cell permeant reagent 2′,7′-dichlorofluorescindiacetate (DCFDA) (Sigma), a fluorogenic dye that measures hydroxyl, peroxyl, and other ROS activity within the cell. After diffusion into the cell, DCFDA is deacetylated by cellular esterases to a non-fluorescent compound, which is later oxidized by ROS into 2′,7′-dichlorofluorescein (DCF). DCF is a highly fluorescent compound, and it has been detected by flow cytometry analysis using CyAn ADP flow cytometer (Beckman Coulter`), and data were analyzed with FCS5 Express software (De Novo Software).

### Immunofluorescence analysis

To describe the morphological features of CD44v8-10^pos^ and CD44v8-10^neg^ cells, immunofluorescence experiments, followed by confocal microscopy analysis, were performed. Briefly, cells cultured for 24 h in 10% FBS on Ibidi slides (μ-Slide 8 well, cat. 80826; Ibidi, Gräfelfing, Germany) were fixed in 4% paraformaldehyde in PBS (pH 7.4) at 4 °C for 10 min. Then, cells were permeabilized in PBS 1% BSA 0.1% Triton for 1 h and incubated overnight with anti-vinculin antibody (Cat sc-73614, 1:50 dilution; Santa Cruz Biotechnology). Samples were washed 3 times in PBS/BSA/Triton for 30 min and incubated with the appropriate secondary antibody: FITC-conjugated donkey anti-mouse (Jackson ImmunoResearch, Ely, UK), TOPRO-3 Iodide for nuclei staining and rhodamine phalloidin (1:40 dilution, Molecular Probes, Invitrogen, Eugene, OR, USA) for F-actin visualization were used. Then samples were washed 3 times in PBS/BSA and stored in 50 μL of glycerol/PBS pH 9.5 for confocal microscopy analysis. As negative control, the primary antibody was omitted. Immunofluorescence experiments were analyzed using Leica confocal microscope (Laser Scanning TCS SP2 equipped with Kr/Ar and He/Ne lasers; Leica, Mannheim, Germany). Laser lines were 488 and 543 for FITC and TRITC excitations, respectively. The images were scanned under a 20× or 40× oil immersion objective. Optical spatial series with a step size of 1 μm were recovered. Lamellipodia, filopodia, and ruffles were quantified on the maximum projection of series acquired. Colocalization analysis (FITC/green signal) and (TRITC/red signal) were performed by Leica Confocal software.

For immunofluorescence analysis of cytokeratins, anti-pan-cytokeratins (monoclonal mouse anti-human Dako Omnis clone: AE1/AE3; Dako) and Alexa fluor 546-conjugated secondary antibody (ThermoFisher Scientific) were used. Cells were washed in PBS and stained with 600 nM DAPI (ThermoFisher Scientific). Images were acquired by fluorescence light microscope (Zeiss Axioskop 2 plus; Zeiss, Jena, Germany).

### Adhesion assay

Under sterile conditions, 2 × 10^5^ cells were seeded for 90 min on 12-well plates pre-coated with 10 μg/mL of type I collagen at 37 °C. After removing the culture medium, the adherent cells were washed with PBS for 3 times and fixed in 4% paraformaldehyde (Electron Microscopy Sciences, Hatfield, PA, USA) in PBS 10 min at room temperature. The adherent cells were incubated with crystal violet for 30 min at room temperature and washed distilled water for 3 times. The plates were left to dry overnight. The following day, 10% acetic acid was added to each well to solubilize the crystal violet, and the plates were read by spectrophotometer at 550 nm.

### Enzyme-linked immunoassay

For quantification of secreted IL-6, medium from CD44v8-10^pos^ and CD44v8-10^neg^ cells conditioned (CM) for 48 h was assayed by enzyme-linked immunoassay (ELISA) (R&D Systems Minneapolis, MN), according to the manufacturer’s instruction. Ninety-six-well plates (Nunc, Milan, Italy) were coated with 4 μg/mL rabbit polyclonal anti-IL6 antibody (clone: H-121; Santa Cruz Biotechnology) in 100 μL/well of PBS and incubated overnight at 4 °C. After 3 washes with PBS, 100 μL/well of blocking solution (PBS containing 0.5% BSA) was added at room temperature for 1 h. After 3 washes with PBS, 4 μg/mL of a mouse anti-rabbit horseradish peroxidase (HRP)-conjugated secondary antibodies (clone 5A6; Abcam) were added to each well and incubated for 1 h at 37 °C. After the final 3 washes with PBS, the reaction was developed with Blue POD for 15 min (Roche Applied Science, Milan, Italy) and blocked with 4 N H_2_SO_4_ stop solution, and optical densities were recorded at 450 nm.

### Western blot

Total protein extraction was performed by homogenizing cells lysis buffer containing 1× protease and phosphatase inhibitors cocktail (lysis buffer 10×; Cell Signaling Technology, Danvers, MA, USA). After sonication, the homogenates were centrifuged at 300 g for 5 min at 4 °C. Protein concentrations were determined using BCA (bicinchoninic acid assay) to measure the concentration of protein in a solution. Lysates obtained from 2 subpopulations were analyzed in denaturing condition by SDS-PAGE and transferred on to nitrocellulose membranes (Amersham Bioscience, Little Chalfont, UK). For the experiments with CD44v8-10-knocked-down PC3 cells, the total lysates were prepared 72 h after siRNA/scramble transfection. Membranes were incubated with primary antibodies rabbit anti-human-phospho-β catenin (Ser 33/37 Thr 41) and rabbit anti-human total β catenin, rabbit anti-human N-cadherin, rabbit anti-human P-PARγ, and rabbit anti-human RUNX2 (all from Cell Signaling Technology), and rabbit anti-human MMP-13 (Abcam, Cambridge, UK) followed by goat anti-Rabbit IgG H + L HRP-conjugated secondary antibody (Bio-Rad, Hercules, CA, USA) and visualized by ECL (Western nova 2.0; Cyanagen, Bologna, Italy) using Chemidoc Gel Imaging System Image Lab version 5.2.1. software (Bio-Rad). Densitometric analysis of immunoblots was performed by ImageJ.

### Osteoblast culture

Bone fragments were obtained from healthy subjects who underwent femoral surgery for traumatic fractures. Bone fragments were subjected to sequential digestion with type IV collagenase (Sigma-Aldrich) and trypsin (Gibco). Cells obtained with this method were evaluated for alkaline phosphatase (ALP) activity (kit #86; Sigma-Aldrich) and expression of osteoblast markers.

### PC3 mineralization assay

CD44v8-10^pos^ and CD44v8-10^neg^ PC3 cells were cultured in RPMI plus 10% FBS, 10 mmol/L β-glycerophosphate, and 50 μg/mL ascorbic acid to induce mineralization. After 3 weeks, von Kossa and Alizarin Red staining were performed to evaluate the mineralized area by an image analysis system (NIS-Elements BR 4.50.00).

### Treatment of bone cells with CM isolated from PC3 cells

CM were collected from CD44v8-10^pos^ and CD44v8-10^neg^ PC3 cells grown in RPMI plus 10% FBS until 80% confluence. The media were then replaced with serum-free medium containing BSA 0.25%, and after 48 h, supernatants were harvested and stored at −20 °C until use. For the experiments employing PC3 treated with SiRNAs targeting CD44v8-10 or scramble RNA, CM were collected 72 h after transfection and after being confirming (> 70% CD44v8-10 down-regulation by flow cytometry analysis.

For osteoclastogenesis, PBMC of healthy donor were diluted in PBS and layered over Ficoll 1.077 g/mL (PANCOLL; PAN Biotec, Aidenbach, Germany) and centrifuged at 400 *g* for 30 min. “Buffy coat” was collected and washed twice with PBS. Cells were resuspended in Dulbecco’s modified Eagle medium containing 50 U/mL penicillin, 50 mg/mL streptomycin, 2 mM L-glutamine and 10% FBS (ThermoScientific). Then 1 × 10^6^ cells/cm^2^ were plated on a 96-well plate, and after 3 h, cell cultures were rinsed to remove non-adherent cells. Cells were cultured in the presence of 20 ng/mL M-CSF and 30 ng/mL RANK-L or 50% CM obtained from PC3 cultures without osteoclastogenic cytokines. Medium was replaced every 3–4 days. After 14 days, cells were eventually fixed in paraformaldehyde and stained for tartrate-resistant acid phosphatase (TRAcP) and DAPI to evaluate count TRAcP-positive multinucleated (> 3 nuclei) cells.

For osteoblast experiments, control osteoblasts were cultured on 96-well plates and treated for 48 h with 100% CM obtained from PC3 cultures or 0.25% BSA as control.

### Statistical analysis

All experiments were repeated at least 3 times. All numerical data were described as mean ± standard error of mean. Data were analyzed using 2-tailed Student’s *t*-test. A *P*-value ≤0.05 was considered significant.

## Results

### CD44v8-10^pos^ PC3 cells express an epithelial phenotype and increased levels of aggressiveness markers

**Figure 1:**
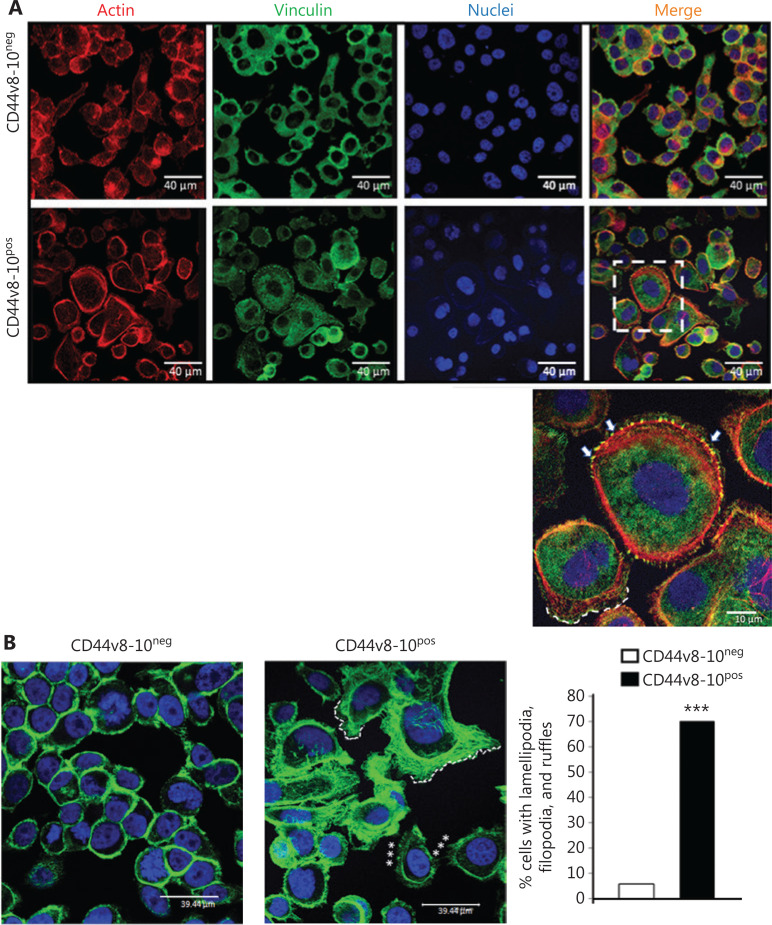
Representative images of confocal microscopy analysis of actin (red) and vinculin (green) in CD44v8-10^pos^ and CD44v8-10^neg^ PC3 cells. (A) In CD44v8-10^neg^, vinculin appears as a diffuse signal in cytoplasm of the cells and partially co-localizes with actin (merge). In CD44v8-10^pos^, vinculin is well evident also in the membrane protrusion (selected area). Scale bar: 40 μm. Magnification of selected area shows filopodia (arrows) in which vinculin co-localizes with actin filaments to form focal adhesions. Dashed line indicates lamellipodia frequently observed in these cells. Scale bar: 10 μm. TO-PRO3 (blue) was used for nuclear staining. (B) Representative confocal images of F-actin distribution in CD44v8-10^neg^ cells and in CD44v8-10^pos^ cells. In CD44v8-10^pos^ cells are evident lamellipodia (dotted lines), filopodia*, and ruffles. Scale bars: 39 and 44 μm. Right panel shows the percentage of cells presenting lamellipodia, filopodia, and ruffles in both populations. ***P < 0.001, Student’s paired *t*-test.

In a previous study, we isolated a minor subpopulation of CD44-negative cells within the highly aggressive PC cell line PC3 that, when cultured, these CD44-negative cells rapidly converted to a CD44-positive phenotype expressing specifically the CD44v8-10 isoform (CD44v8-10^pos^), displayed stem cell-like features and showed a marked invasive phenotype *in vitro* that was lost by silencing the CD44 variant^3^. These traits prompted us to further investigate the molecular and functional features that make the CD44v8-10^pos^ population so highly invasive. To study the cytoskeletal remodeling involved in the increased invasive potential of CD44v8-10^pos^ cells, we investigated the intracellular distribution of F-actin and vinculin in CD44v8-10^pos^ and CD44v8-10^neg^ cells. Comparing the 2 populations by confocal analysis, we found an abundance of lamellipodia in CD44v8-10^pos^ cells consistent with a stronger migratory and invasive phenotype, compared to CD44v8-10^neg^ cells (**Figure 1A**). We performed quantitative analyses on images recorded by confocal microscopy, and we found that approximately 70% of CD44v8-10^pos^ cells showed an abundance of lamellipodia, filopodia, and ruffles compared to CD44v8-10^neg^ cells, in which only 5.6% showed these characteristics (**Figure 1B**). The migratory/invasive phenotype is characterized by cytoskeletal rearrangement and involves new focal adhesions (FAs). Vinculin is highly expressed in FAs, where it colocalizes with the actin filaments and recruits proteins involved in FAs dynamics. Accordingly, FAs were specifically found in CD44v8-10^pos^ cells, as highlighted in the magnification of **Figure 1A**.

**Figure 2:**
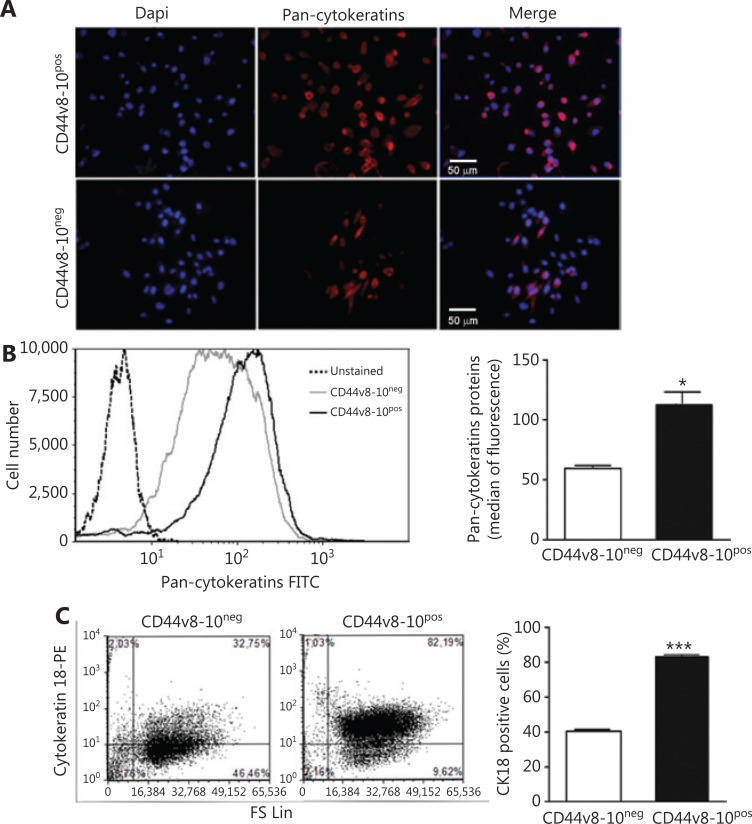
Cytokeratin expression in PC3 subpopulations. (A) Representative immunofluorescence assay by using DAPI for nuclei staining (blue signal) and antibody for pan-cytokeratins (rhodamine, red signal). (B) Flow cytometry analysis using antibodies for pan-cytokeratins and (C) for CK18. Mean ± standard error of mean derived from the value of the median fluorescence of 3 independent experiments (B, right) and from the percentage of CK18-positive cells (C, right) of 3 independent experiments. **P < 0.01; ***P < 0.001, Student’s paired *t*-test. (B and C, left) Representative experiments of flow cytometry analysis for indicated cytokeratins.

**Figure 3:**
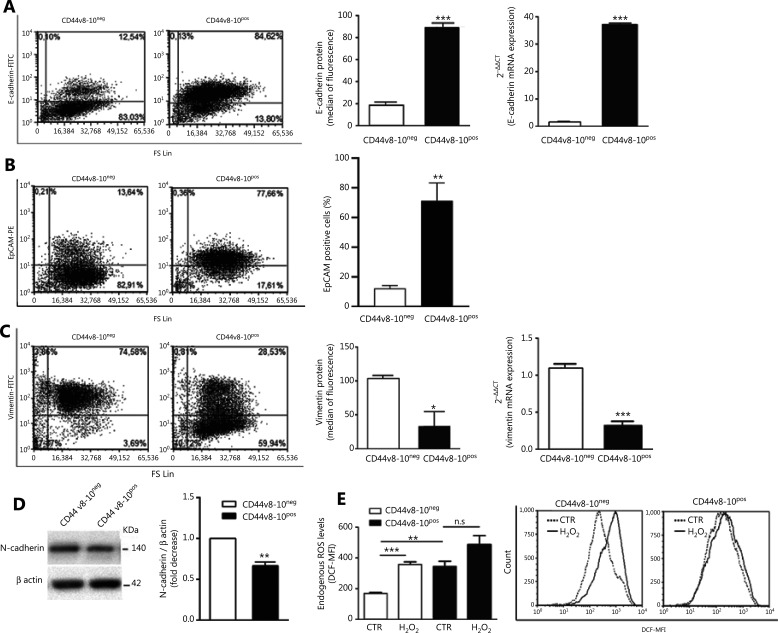
Expression of epithelial/mesenchymal markers and reactive oxygen species (ROS) production in PC3 subpopulations. CD44v8-10^pos^ cells show high levels of E-cadherin (A) and EpCAM (B) and low levels of vimentin (C) and N-cadherin (D) compared with CD44v8-10^neg^ cells. (Middle panels) Histograms of E-cadherin (A), EpCAM (B), and vimentin (C) of 3 independent experiments, and the respective representative flow cytometry plots are shown in the left panels of A, B, and C. The levels of E-cadherin (A, right) and vimentin (C, right) mRNA assayed by real-time quantitative polymerase chain reaction analysis mirror the protein levels. (D, left) Whole-cell extracts of CD44v8-10^pos^ and CD44v8-10^neg^ PC3 cells were analyzed by Western blot for N-cadherin. β-Actin was used as control for equal amounts of proteins loaded. Each blot is representative of three experiments. (D, right) Histogram of the densitometric analysis of the Western blot of 3 separate experiments representing the fold decrease of the N-cadherin/β-actin ratio (CD44v8-10^neg^ value set as 1). (E) Endogenous ROS levels were measured by flow cytometry using DCFDA dye fluorescence in cells treated with or without 200 μM hydrogen peroxide (H_2_O_2_) for 30 min. The graph presents 3 independent experiments and a representative flow cytometry histogram. *P < 0.05; **P < 0.01; ***P < 0.001, Student’s paired *t*-test.

Although it is now widely accepted that activation of the EMT program is fundamental in enabling metastatic dissemination, the specific CD44v8-10 isoform was initially called epithelial CD44 (E-CD44) because it is produced by epithelial-type splicing factor ESRP1 activity, typically expressed in epithelial cells^38^. Accordingly, we have previously shown a very high ESRP1 expression and concomitant low levels of the mesenchymal marker ZEB1 in CD44v8-10^pos^ PC3 cells^3^. On this basis, we set out to further investigate different molecular markers linked to epithelial and mesenchymal phenotype in CD44v8-10^pos^ and CD44v8-10^neg^ PC3 cells. First, we tested the expression of cytokeratins, the intermediate filament components specific of epithelial cells. Using an anti-pan-cytokeratins antibody in immunofluorescence (**Figure 2A**) and flow cytometry (**Figure 2B**) assays, we observed upregulation of cytokeratins in CD44v8-10^pos^ cells, among which a specific antibody showed the prominence of cytokeratin 18 (CK18) (**Figure 2C**), known to correlate with progression of clinical staging in several tumors^39^. Moreover, the CD44v8-10^pos^ population exhibited higher levels of the epithelial markers E-cadherin, both at mRNA (**Figure 3A**, right panel) and protein level (**Figure 3A**, left panels), and EpCAM protein (**Figure 3B**) compared to CD44v8-10^neg^ cells. On the contrary, the mesenchymal markers vimentin (**Figure 3C**) and N-cadherin (**Figure 3D**) were expressed at a lower extent in CD44v8-10^pos^ cells than in CD44v8-10^neg^ cells.

It has been demonstrated in breast cancer and PC that the most aggressive and metastatic cells showed high levels of ROS^9,40^. Based on this evidence, we analyzed the ROS levels in CD44v8-10^pos^ and CD44v8-10^neg^ PC3 cells by flow cytometry analysis using DCFDA, a widely used ROS indicator. As shown in **Figure 3E**, CD44v8-10^pos^ cells had higher ROS basal levels than the negative population. Treatment with H_2_O_2_ significantly increased the ROS levels only in CD44v8-10^neg^ cells, showing that CD44v8-10^pos^ cells are equipped to better respond to oxidative stress than CD44v8-10^neg^ cells.

Altogether, our data demonstrate that CD44v8-10^pos^ PC3 cells display a clear epithelial phenotype, surprisingly associated with a great invasive potential.

### Association between CD44v8-10 expression and the prognosis of patients with PC

**Figure 4:**
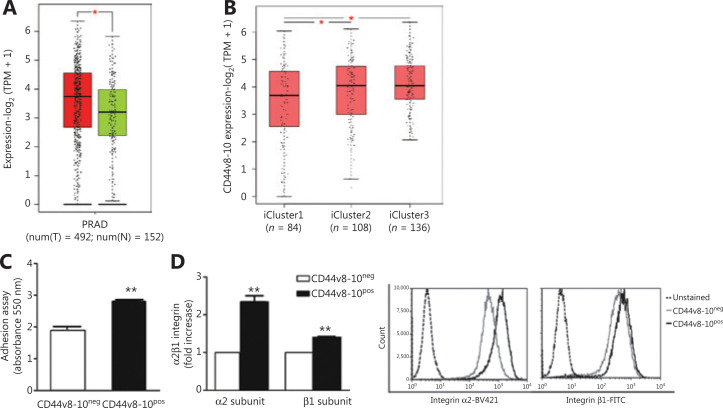
Association between CD44v8-10 expression and the prognosis of patients with prostate cancer (PC). Adhesion ability to type I collagen of PC3 cell populations. (A) CD44v8-10 expression (ENST00000433892.6) was analyzed using GEPIA software in normal/benign samples (from TCGA and GTEx, green bar) and in prostate adenocarcinoma (PRAD from TCGA, red bar). Cancer tissue samples (*n* = 492) and normal tissues (*n* = 152). *P < 0.05, Student’s *t*-test. (B) Analysis of patients with PC collected by the TCGA dataset shows CD44v8-10 expression in the iClusters: iCluster1, mild prognosis; iCluster2, intermediate prognosis; iCluster3, poor prognosis. (C) The 2 PC3 subpopulations were cultivated on type I collagen-coated plates for 90 min. After staining with crystal violet, the cells were detached and the absorbance was determined using a spectrophotometer. (D) Flow cytometry analysis for α2β1-integrin, type I collagen receptor, and the relative representative flow cytometry histogram. Data represent the mean ± standard error of mean derived from 3 independent experiments. *P < 0.05; **P < 0.01; Student’s paired *t*-test.

To strongly correlate our *in vitro* results with PC progression, we interrogated The Cancer Genome Atlas (TCGA) dataset analyzing CD44v8-10 expression in 492 PC patient tissues *vs.* 152 non-tumoral prostate tissues^41^. The results showed that CD44v8-10 expression level was significantly higher in PC tissues (**Figure 4A**), corroborating our *in vitro* results. Interestingly, using Gene Expression Profiling Interactive Analysis (GEPIA), a web-based tool to study the distribution of CD44 isoform expression^42^, the violin plot showed that CD44v8-10 (ENST00000433892.6) was the CD44 isoform most expressed in PC tissues (**Supplementary Figure S1**). To further address the role of CD44v8-10 in PC prognosis, we analyzed the CD44v8-10 expression in the iClusters by the TCGA dataset^41^. We reported that CD44v8-10 expression was significantly higher in the iCluster3, which represents the group with the poorest prognosis, including the gene signatures most associated with the clinical and molecular features of patients with metastases [dramatic alterations in patient genomes such as fusions of androgen-regulated promoter with ERG and other members of the E26 transformation-specific (ETS) family of transcription factor] (**Figure 4B**).

Overall, these findings on PC tissues indicate the oncogenic role of CD44v8-10 in maintaining the tumor phenotype and in metastasis.

### CD44v8-10 drives osteomimetic features in PC3 cells, favoring bone-specific metastasis

Starting from data observed in PC patients, we performed *in vitro* experiments aiming to highlight the involvement of CD44v8-10 in bone metastasis. It is postulated that the activation of EMT during cancer progression enables cancer cells to acquire migratory, invasive, and stem-like properties^17^; nevertheless, once the tumor cells arrive at metastatic sites, they re-express epithelial markers to perform metastatic colonization^43^. Therefore, considering the derivation of PC3 cells from bone metastases, we can suppose that the epithelial phenotype of CD44v8-10^pos^ cells may account for their great ability to metastasize to the bone. To address this hypothesis, we examined the osteotropic effect of both CD44v8-10^pos^ and CD44v8-10^neg^ cells investigating their capability to adhere to type I collagen, the most abundant component of organic extracellular matrix in the bone tissue. By colorimetric assay, we observed that CD44v8-10^pos^ cells showed higher ability to adhere to type I collagen compared with the CD44v8-10^neg^ population (**Figure 4C**). In line with this finding, CD44v8-10^pos^ cells strongly expressed also α2β1-integrin, which represents the type I collagen receptor, reported to be a prostate CSC marker (**Figure 4D**). These features could facilitate the metastatic colonization of CD44v8-10^pos^ cells within the bone tissue.

**Figure 5:**
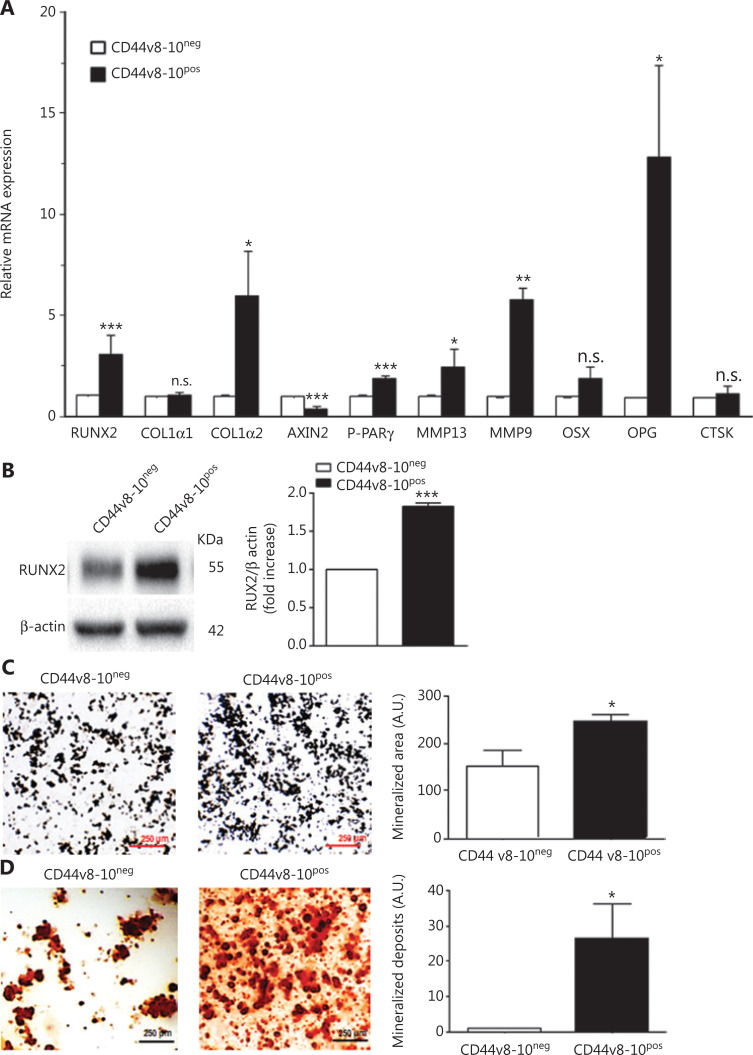
Expression analysis of genes involved in osteomimicry process and functional assay for osteoblast-like activity. (A) Real-time quantitative polymerase chain reaction analysis for genes encoding proteins involved in osteoblast and osteoclast differentiation and function. β-actin was used as an internal control. (B, left) Whole-cell extracts of CD44v8-10^pos^ and CD44v8-10^neg^ PC3 cells were analyzed by Western blot for RUNX2. β-actin was used as control for equal amounts of proteins loaded. Each blot is representative of 3. (B, right) The histogram represents the densitometric analysis of Western blot of 3 separate experiments representing the fold increase of the RUNX2/β-actin ratio (CD44v8-10^neg^ value set as 1). (C, left) CD44v8-10^pos^ and CD44v8-10^neg^ PC3 cell ability to release mineralized nodules revealed by von Kossa staining and (D, left) to form calcium deposits revealed by Alizarin Red staining. (C and D, right) Mean ± standard error of mean derived from 3 independent experiments performed as described in the left. *P < 0.05; **P < 0.005; ***P < 0.001 *vs.* CD44+, Student’s paired *t*-test.

It is well known that several human carcinomas, in particular PC, can acquire phenotypic aspects of bone cells (“osteomimicry” process) to avoid detection by the immune system and establish colonies in the bone microenvironment^18^. Osteomimetic features adopted by cancer cells involve the expression of bone-related molecules such as osterix, osteoprotegerin (OPG), runt-related transcription factor (RUNX2), metalloprotease (MMP) 9, MMP-13, and cathepsin K^44,45^. We examined the expression of these proteins by real-time PCR (qPCR) in CD44v8-10^pos^ and CD44v8-10^neg^ cells to investigate the osteomimetic phenotype of these PC3 subpopulations. As shown in **Figure 5A**, we found that CD44v8-10^pos^ expressed the highest levels of RUNX2, a2 type I collagens, master genes of osteoblast differentiation and function, as well as of OPG, MMP-9, MMP-13, and P-PAR-γ, which implicated in bone resorption^46^. Conversely, in CD44v8-10^pos^ cells, we observed a reduction of Axin2, an inhibitor of bone formation, consistent with the increase of RUNX2. The higher expression of the osteogenesis master gene *RUNX2* in CD44v8-10^pos^ cells was confirmed also at protein levels by Western blot (**Figure 5B**). Moreover, a higher osteoblastic-like behavior of CD44v8-10^pos^ compared with CD44v8-10^neg^ cells was demonstrated by their ability to form calcium deposits and to release mineralized nodules revealed by Alizarin Red and von Kossa staining, respectively (**Figure 5C and 5D**).

**Figure 6:**
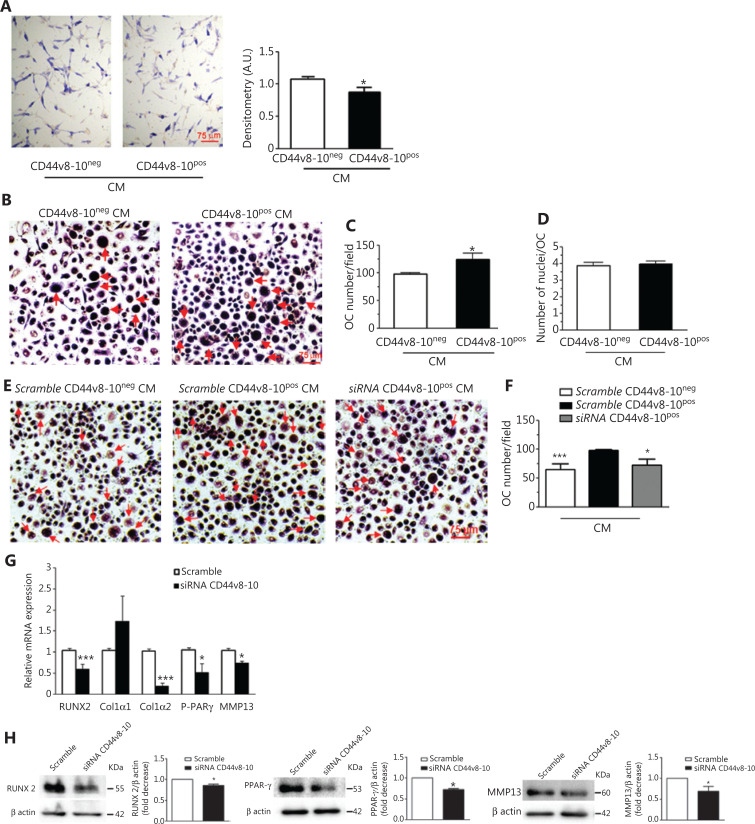
Effects of conditioned medium (CM) from CD44v8-10^pos^ and CD44v8-10^neg^ PC3 cells on osteoblasts and osteoclastogenesis and effect of CD44v8-10 siRNA on osteomimicry genes expressed by CD44v8-10^pos^ PC3 cells. Healthy-donor osteoblasts were treated 48 h with 100% CM obtained from 2 different PC3 cultures and cytochemical analysis of alkaline phosphatase (ALP) activity of osteoblasts was performed. Representative ALP staining (A, left) and densitometric analysis of ALP staining of 3 independent experiments evaluated as arbitrary units (A.U.) (A, right). Results are expressed as mean ± standard error of mean (SEM). *P < 0.05. (B) Healthy-donor peripheral blood mononuclear cells (PBMCs) were treated with 50% CM obtained from 2 different PC3 cultures and TRAcP staining was performed. In the representative images, the osteoclasts are indicated by red arrows. (C) Number of multinucleated (> 3 nuclei) TRAcP-positive osteoclasts (OC) and (D) number of nuclei per osteoclast of 3 independent experiments were evaluated. Results are expressed as mean ± SEM. *P < 0.05. (E) Three healthy-donor PBMCs were treated with 50% CM obtained from 3 different PC3 cultures treated with CD44v8-10 siRNA/scramble and TRAcP staining was performed. In the representative images the osteoclasts are indicated by red arrows. (F) Histograms show number of multinucleated (> 3 nuclei) TRAcP positive osteoclasts (OC) in the experimental setting described in panel E. Results are expressed as mean ± SEM. *P < 0.05; ***P < 0.001 *vs.* scramble CD44v8-10^pos^-derived CM treatment. Effect of CD44v8-10 siRNA on the expression of the indicated osteomimicry genes at mRNA (G) and protein (H) levels. Each Western blot is representative of three experiments. β-actin was used as control for equal amounts of proteins loaded. The histograms show the densitometric analysis of Western blot of 3 separate experiments representing the relative expression being scramble RNA value set as 1. *P < 0.05; ***P < 0.001.

To test whether soluble factors secreted by CD44v8-10^pos^ cells are involved in their higher bone remodeling ability, we used CM derived from the 2 PC3 cell populations in functional assays assessing osteoclastogenesis and osteoblast formation. First, healthy-donor osteoblasts were treated with 100% CM obtained from 2 different cultures of the 2 PC3 subpopulations or medium plus 0.25% BSA. As shown in **Figure 6A**, a reduction of ALP activity was revealed in osteoblasts treated with CM from CD44v8-10^pos^ PC3 cells, suggesting the ability of these cells to weaken the function of normal osteoblasts. Then, healthy-donor PBMCs were treated with 50% CM obtained from 2 different cultures of the 2 PC3 subpopulations without adding osteoclastogenic cytokines and the effects on osteoclastogenesis were determined by analyzing the osteoclast specific TRAcP staining and by counting the number of TRAcP-positive multinucleated (≥3 nuclei) cells. Osteoclastogenesis was significantly induced by CM derived from CD44v8-10^pos^ PC3 cells compared with that from CD44v8-10^neg^ PC3 cells (**Figure 6B and 6C**), whereas no differences were observed in the number of nuclei per osteoclast (**Figure 6D**). These findings are consistent with the fact that bone lesions resulting from PC are primarily osteosclerotic, but are also associated with increased bone resorption^20^.

To assess the functional relationship between CD44v8-10 and osteoclast differentiation, we tested in osteoclastogenesis assay CM derived from CD44v8-10^pos^ cells knocked down by RNA interference using a specific siRNA, targeting v9 exon. The results in **Figure 6E and 6F** show that CM derived from CD44v8-10 siRNA cells significantly reduced the number of differentiated osteoclasts induced by scramble CD44v8-10^pos^ cell-derived CM, almost reaching up to the osteoclastogenesis rate observed with CM derived from CD44v8-10^neg^ PC3 cells, highlighting the role of this variant in osteonecrotic metastasis.

### CD44v8-10 knock-down impairs osteomimicry in PC3 cells

Moreover, we examined the role of CD44v8-10 in osteomimetic gene expression in CD44v8-10^pos^ PC3 cells by real-time PCR and Western blot analyses. Knock-down of CD44v8-10 by siRNA significantly reduced the expression of RUNX2, type I a2 collagen, involved in osteogenesis, as well as of MMP-13, P-PAR-γ involved in bone resorption (**Figure 6G**), suggesting that the specific variant CD44v8-10 plays a role in mediating upregulation of osteomimetic genes in PC3 cells. Similarly, CD44v8-10 siRNA induced a significant decrease in the expression of RUNX2, MMP-13, and P-PAR-γ proteins (**Figure 6H**). Flow cytometry analysis shows the silencing effect > 70% at 12.5 nM siRNA on CD44v8-10 protein level (**Supplementary Figure S2**).

### CD44v8-10 upregulates osteomimetic genes through the activation of Wnt/TAZ signaling pathways

**Figure 7:**
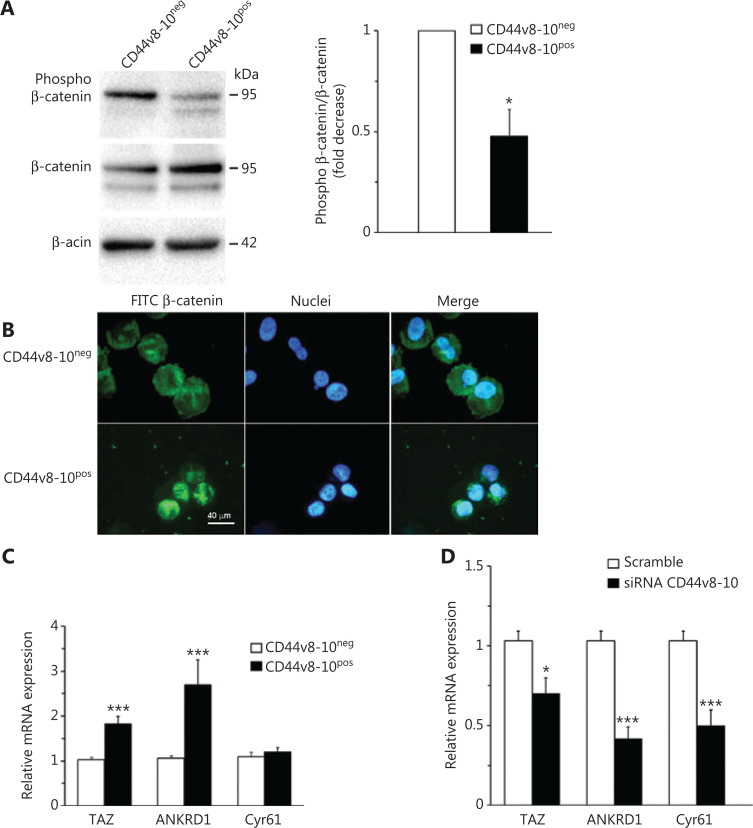
Involvement of Wnt and TAZ pathways in CD44v8-10-mediated signaling. Whole-cell extracts of CD44v8-10^pos^ and CD44v8-10^neg^ PC3 cells were analyzed by Western blot for Phospho-β-catenin and total-β-catenin (A, left). β-Actin was used as control for equal amounts of proteins loaded. Each blot is representative of 3. The histograms represent the densitometric analysis of Western blot of 3 separate experiments representing the fold decrease (CD44v8-10^neg^ PC3 cells *vs.* CD44v8-10^pos^ PC3 cells) of the phospho-β-catenin/β-catenin ratio (A, right). (B) Representative immunofluorescence assay by using DAPI for nuclei staining (blue signal) and antibody for β-catenin (fluorescein, green signal). (C) TAZ and its targets expression levels were evaluated by real-time quantitative polymerase chain reaction in the 2 PC3 cell populations in basal conditions and (D) in CD44v8-10^pos^ PC3 cells after treatment with CD44v8-10 siRNA. Results are expressed as mean ± standard error of mean. *P < 0.05; ***P < 0.001.

Since it is known that Wnt signaling positively regulates tumor progression and osteomimicry in PC^47^ and that CD44 acts as an inducer of the Wnt-dependent β-catenin signaling pathway^31^, to identify the possible mechanism underlying CD44v8-10-mediated osteomimetic traits, we examined the constitutive level of Wnt activation in CD44v8-10^pos^ and CD44v8-10^neg^ PC3 cells. Western blot analysis in **Figure 7A** shows a decreased level of phosphorylated (inactive) β-catenin in CD44v8-10^pos^ cells compared to CD44v8-10^neg^ cells, suggesting that CD44v8-10 is a positive regulator of canonical Wnt signaling. Higher nuclear localization of total β-catenin in CD44v8-10^pos^ cells assayed by immunofluorescence indicates an activation of Wnt pathway in this cell population (**Figure 7B**). TAZ-dependent signaling is another pathway involved in metastatic potential and bone metastasis that could represent a possible intermediate in CD44v8-10/Wnt/osteomimetic genes^48^ or a novel mediator activated by CD44v8-10. To verify this hypothesis, we measured the expression levels of this factor and its well-known ANKRD1 and CYR61 targets genes by RT-qPCR. We observed a higher expression of ANKRD1 in CD44v8-10^pos^ PC3 cells than in CD44v8-10^neg^ cells, whereas no difference was observed in Cyr61 expression between the 2 cell populations (**Figure 7B**). Knock-down of CD44v8-10 by siRNA reverted the increased levels of TAZ and ANKRD1 and also reduced Cyr61 expression (**Figure 7C**), showing for the first time a role of a CD44 variant in TAZ-mediated pathway.

### CD44v8-10^pos^ cells produce high levels of IL-6 and express the immunosuppressive protein PD-L1

**Figure 8:**
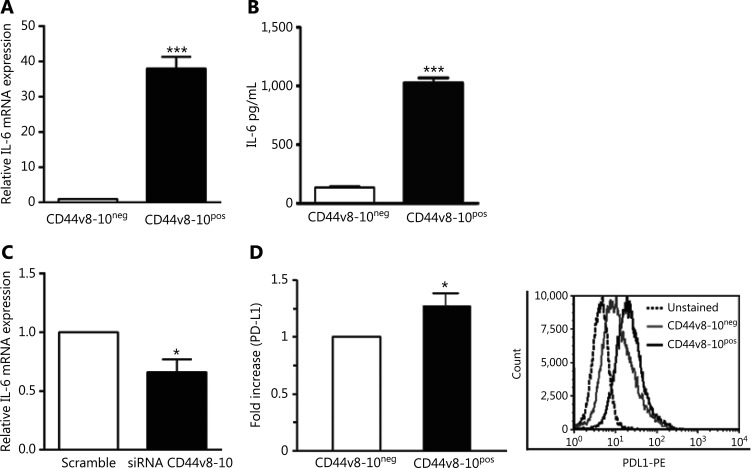
CD44v8-10^pos^ cells produce high levels of interleukin (IL)-6 and express the immunosuppressive protein PD-L1. (A) Real-time quantitative polymerase chain reaction (RT-qPCR) analysis for IL-6 mRNA in the 2 PC3 populations (CD44v8-10^neg^ value set as 1). (B) Enzyme-linked immunoassay for IL-6 in medium conditioned for 48 h from CD44v8-10^pos^ and CD44v8-10^neg^ PC3 cells. (C) RT-qPCR analysis for IL-6 mRNA in CD44v8-10^pos^ PC3 cells after treatment with CD44v8-10 siRNA (scramble value set as 1). (D, left) Flow cytometry evaluation of the percentage of cells expressing membrane PD-L1 in the 2 PC3 cell populations. (D, right) Representative flow cytometry histogram. Data represent the mean ± standard error of mean derived from 3 independent experiments. *P < 0.05; ***P < 0.001, Student’s paired *t*-test.

Since IL-6 is a cytokine that plays an important role in bone metastasis formation and promotes osteolytic bone metastasis by increasing osteoclast activity^49^, we examined the levels of IL-6 in our 2 PC3 subpopulations and observed higher levels of IL-6 mRNA in CD44v8-10^pos^ cells compared to CD44v8-10^neg^ cells (**Figure 8A**). Consistent with mRNA expression analysis, CD44v8-10^pos^ cells secreted markedly higher levels of IL-6 protein, detected in the culture medium conditioned for 48 h, as shown by ELISA (**Figure 8B**), corroborating the osteoclastogenic capability of CD44v8-10^pos^ PC3 cells. A significant reduction of IL-6 expression was observed in CD44v8-10^pos^ PC3 cells treated with CD44v8-10 siRNA, confirming the role of this CD44 variant on osteolytic bone metastasis formation (**Figure 8C**).

Besides its role in osteoclastogenesis, IL-6 signaling acts in bone microenvironment also in immunosuppressive network resulting in impaired adaptive immune responses against the tumors^23^. Therefore, we analyzed the presence of the membrane-bound immunosuppressive molecule PD-L1 by flow cytometry. Ligation of programmed cell death (PD-1) on tumor-specific T cells with its ligand, PD-L1, expressed on tumor cells, is involved in tumor-induced immunosuppression^50^. **Figure 8D** shows that the membrane expression of PD-L1 protein is higher in CD44v8-10^pos^ cells than in CD44v8-10^neg^ cells.

## Discussion

Despite the clinical relevance of bone metastasis, the molecular mechanisms that underlie the bone tropism of PC are not well understood. This gap in knowledge is due in part to difficulties in obtaining metastatic bone tissue from patients^51^; therefore, the PC3 cell line, derived from human PC bone metastasis, is widely used to study the mechanisms underlying bone metastasis aggressiveness. Our data show that CD44v8-10^pos^ PC3 cells display markedly higher epithelial and osteomimetic features compared to the CD44v8-10^neg^ population and are thus more capable of forming bone metastasis and creating an immunosuppressive microenvironment in bone. Our study suggest that CD44v8-10 may not simply be a marker of PC more aggressive metastatic cells, but is also actively involved in the progression of the disease. Notably, we have disclosed a novel pathway activated by this specific CD44 variant isoform, involving the transcriptional factor TAZ and likely the molecular Wnt/TAZ axis.

Surprisingly, we found a frank epithelial phenotype in the more aggressive CD44v8-10^pos^ PC cells with a highest levels of the epithelial markers cytokeratins, EPCAM, and E-cadherin associated to an invasive phenotype characterized by abundance of lamellipodia and FA structures. Interestingly, it has been demonstrated in PC that E-cadherin^pos^ cells more efficiently proliferate and form colonies of holoclonal-type morphology than E-cadherin^neg^ cells; E-cadherin^pos^ PC3 and DU145 cells are subsets of cells retaining stem cell functionality and are therefore able to form metastatic colonies at distant sites^52^. Moreover, a recent work^53^ shows that E-cadherin is up-regulated at the periphery of PC3 and DU145 3D-spheroids, indicating a stronger cohesion among the front cells of the migrating epithelial sheet to ensure tissue integrity during collective cell migration^54^. A similar E-cadherin expression pattern was previously described in pancreatic adenocarcinoma cells^55^. Recent studies suggest that because of epithelial plasticity, tumor cell phenotypes cannot be simply classified as epithelial or mesenchymal, but a spectrum of intermediate states should be considered^56^. Based on these findings, it is emerging that the canonical point of view whereby mesenchymal traits attest the highest aggressiveness needs to be re-considered and warrants further investigation^57^. We also show that CD44v8-10^pos^ PC cells have higher ROS basal levels than the negative population. Other studies have shown that the aggressive growth and metastatic ability of PC cells result from the high levels of intracellular ROS generated in these cells, which are very critical for the maintenance of malignant phenotype^40,58^. Moreover, in metastatic breast cancer, only CD44v8-10-expressing cells are able to invade lung site rich of ROS and to establish metastatic lesions^9^.

Since, in PC, MET is an important process for cancer cell re-differentiation and metastatic colonization into the bone, we investigated whether CD44v8-10^pos^ PC3 cells can efficiently adhere to the bone. We assessed that CD44v8-10^pos^ population adhered more to type I collagen than CD44v8-10^neg^ cells and showed up-regulation of type I collagen receptor α2β1-integrin. This integrin is the prevalent cell-surface protein and has the most relevant role in the invasion of PC3 cells when compared with other integrins^59,60^. The binding of CD44 to osteopontin, a glycoprotein of bone matrix, is β1-integrin-dependent and leads to enhanced cell motility and chemotaxis^61^. Moreover, several candidate markers of prostate stem cells have been proposed, including α2β1-integrin^62^.

These findings prompted us to hypothesize that CD44v8-10 could represent a biological marker to identify a highly aggressive PC bone metastatic population. By RT-qPCR, we demonstrated that CD44v8-10^pos^ cells express significantly higher levels of pivotal genes involved in osteoblast and osteoclast differentiation compared with CD44v8-10^neg^ cells: these data strongly suggest a high ability of PC to form both osteosclerotic and osteolytic metastasis. Importantly, a higher osteoblastic-like behavior of CD44v8-10^pos^ compared with CD44v8-10^neg^ cells was demonstrated by their ability to release mineralized nodules.

Additionally, since cancers that metastasize to bone perturb the remodeling process through a series of soluble factors acting on osteoclasts and osteoblasts, we tested the effect of CM derived from the 2 PC3 subpopulations on bone cells. Notably, we observed that osteoclastogenesis was significantly induced by CM derived from CD44v8-10^pos^ PC3 cells compared with that from CD44v8-10^neg^ PC3 cells, and the effect was totally reverted if we used CM derived from CD44v8-10-knocked-down cells, providing evidence of the involvement of CD44v8-10 in osteoclast differentiation.

Conversely, we observed that CM derived from CD44v8-10^pos^ cells reduced osteoblast ALP staining, intriguingly suggesting that these tumor cells are able to take control of bone microenvironment impairing the normal bone remodeling process. In the literature, the role of this CD44 variant in the onset of metastasis is unclear because it has been shown that CD44 expression promotes bone metastases in human breast cancer^63^ and that CD44v8-10 and CD44s possess comparable roles in the development of bone metastases in a mouse model of breast cancer^64^. Therefore, to determine whether osteomimetic traits displayed by CD44v8-10^pos^ PC cells were dependent on CD44v8-10 expression, we evaluated the expression of osteomimetic genes in cells knocked down for CD44v8-10 by siRNA. We observed a strong reduction of mRNA and proteins involved both in bone resorption (MMP-13, P-PAR-γ) and bone deposition (RUNX2, type Iα2 collagen), indicating a role of CD44v8-10 both in osteosclerotic and osteolytic lesions. In particular, among the genes modulated by CD44v8-10, it is known that RUNX2 is a transcription factor and master regulator of bone formation highly expressed in tumor cells that metastasize to bone^44^. Accordingly, RUNX2 nuclear localization was found to be up-regulated in PC, and it was suggested that this could be used as a predictor of metastasis in PC^65^. In line with our results, it has been reported that knock-down of CD44 reduced the expression of RUNX2 at mRNA and protein levels in PC3 cells^66^, showing a direct relationship of CD44 expression with RUNX2 activation in PC3 cells. Of note, a recent paper reported that in PC3 cells RUNX2 forms a complex with CD44 intracellular domain as a co-transcriptional factor, activating the expression of metastasis-related genes, such as MMP-9^67,68^, which we found to be up-regulated in CD44v8-10^pos^ cells. However, no specific CD44 isoform has been implicated by these authors; in the present work, by means of CD44v8-10 siRNA, we demonstrate a key role of this variant in RUNX2 expression in PC3 cells.

The use of large-scale clinicogenomic data sets showed that CD44v8-10 isoform is more expressed in prostate tumors than in normal glands, as we reported in our previous paper^3^, and that CD44v8-10 is the CD44 variant most expressed in human PC out of 38 isoforms. In addition, our data were corroborated by TCGA analysis in PC patients showing that CD44v8-10 expression is significantly higher in the iCluster3, which represents the group with poorest prognosis, likely with bone metastasis.

Several lines of evidence clearly indicate that CD44 selects unique downstream effectors that influence multiple cellular functions^69^. To gain insight into the intracellular pathways activated by CD44v8-10 that can be involved in osteomimicry, in our model, we investigated both Wnt and TAZ pathways, which are well known to be involved in bone differentiation and metastasis^70,71^. The functional connection among TAZ, the downstream effector HIPPO pathway, and Wnt signaling in osteoblast differentiation has been reported, and TAZ was identified as a downstream effector of Wnt signaling^48,72^. Moreover, Wnt itself can be activated by CD44 signaling^31^. In this work, we show that both Wnt- and TAZ-dependent signaling are up-regulated in CD44v8-10^pos^ cells compared with CD44v8-10^neg^ cells. Therefore, on the basis of our results, we can speculate that the activation of Wnt by CD44v8-10 results in direct expression of Wnt-mediated osteomimetic genes or that Wnt may activate TAZ, suggesting the axis CD44v8-10/Wnt/TAZ/osteomimetic genes. In contrast with our data concerning high ROS levels in CD44v8-10^pos^ cells, it has been previously demonstrated that, in stem-like cancer cells, high ROS levels can inhibit Wnt pathway through β-catenin phosphorylation^73^. This discrepancy could be explained by cross-talk among different pathways dependent on tumor cell type.

Finally, as the metastatic cells derived from PC alter the bone remodeling process through the release of several factors^74^, we investigated the expression of IL-6, an important cytokine for osteomimicry and tumor progression, in CD44v8-10^pos^ and CD44v8-10^neg^ cells. We observed a stronger expression of IL-6 mRNA, only partially reverted after CD44v8-10 siRNA, and much higher levels of IL-6 protein secreted by CD44v8-10^pos^ cells compared with CD44v8-10^neg^ cells. The role of IL-6 in cancer is complex and includes autocrine and paracrine mechanisms. Many tumors such as prostate, breast, and colon cancer produce large amounts of IL-6 and express the IL-6 receptor, which allows them to respond to IL-6 stimulation in an autocrine manner^22^. Several studies highlight that IL-6 plays an important role in bone metastasis formation and promotes osteolytic bone metastasis by increasing osteoclast activity and that inhibition of the IL-6 receptor directly blocks osteoclast formation *in vitro* and *in vivo*^75^. Besides its role in osteoclastogenesis, IL-6 signaling affects the immunosuppressive network of bone microenvironment inhibiting the maturation of dendritic cells and Th1 differentiation of CD4^+^ T, which results in impaired adaptive immune responses against the tumors^23^. This further role of IL-6 may be effective in our model because we also found higher levels of membrane-bound PD-L1 protein in CD44v8-10^pos^ cells than in CD44v8-10^neg^ cells. PD-L1, which is expressed on many cancer and immune cells, plays an important part in blocking cancer immunity by binding PD-1 and B7.1 (CD80), both of which are negative regulators of T-lymphocyte activation. Binding of PD-L1 to its receptors suppresses T-cell migration and proliferation and secretion of cytotoxic mediators and restricts tumor cell killing^50^. Interestingly, combined blockade of the mutually regulated immunosuppressive activities of IL-6 and PD-1/PD-L1 signals enhances expression of T-cell-attracting chemokines and exerts a synergistic antitumor effect, suggesting an IL6-PD1/PD-L1 cross-talk in the tumor microenvironment^76^.

## Conclusions

Collectively, our findings indicate that CD44v8-10 could represent a biological marker to identify a more aggressive PC bone metastatic population with a driver role in the osteomimetic process. In addition, these CD44v8-10^pos^ cells could be capable of inhibiting anti-cancer cell immunity in bone microenvironment. We have also identified a novel CD44v8-10-dependent pathway involving the pro-tumoral transcription factor TAZ.

## References

[1] Siegel RL, Miller KD, Jemal A (2019). Cancer statistics, 2019. CA Cancer J Clin.

[2] Macedo F, Ladeira K, Pinho F, Saraiva N, Bonito N, Pinto L (2017). Bone metastases: an overview. Oncol Rev.

[3] Di Stefano C, Grazioli P, Fontanella RA, De Cesaris P, D’Amore A, Regno M (2018). Stem-like and highly invasive prostate cancer cells expressing CD44v8-10 marker originate from CD44-negative cells. Oncotarget.

[4] Li H, Chen X, Calhoun-Davis T, Claypool K, Tang DG (2008). PC3 human prostate carcinoma cell holoclones contain self-renewing tumor-initiating cells. Cancer Res.

[5] Underhill C (1992). CD44: the hyaluronan receptor. J Cell Sci.

[6] Matsumura Y, Tarin D (1992). Significance of CD44 gene products for cancer diagnosis and disease evaluation. Lancet.

[7] Ni J, Cozzi PJ, Hao JL, Beretov J, Chang L, Duan W (2014). CD44 variant 6 is associated with prostate cancer metastasis and chemo-/radioresistance. Prostate.

[8] Hu J, Li G, Zhang P, Zhuang X, Hu G (2017). A cd44v(+) subpopulation of breast cancer stem-like cells with enhanced lung metastasis capacity. Cell Death Dis.

[9] Yae T, Tsuchihashi K, Ishimoto T, Motohara T, Yoshikawa M, Yoshida GJ (2012). Alternative splicing of CD44 mRNA by ESRP1 enhances lung colonization of metastatic cancer cell. Nat Commun.

[10] Zhang H, Brown RL, Wei Y, Zhao P, Liu S, Liu X (2019). CD44 splice isoform switching determines breast cancer stem cell state. Genes Dev.

[11] Zhao P, Xu Y, Wei Y, Qiu Q, Chew TL, Kang Y (2016). The CD44s splice isoform is a central mediator for invadopodia activity. J Cell Sci.

[12] Reinke LM, Xu Y, Cheng C (2012). Snail represses the splicing regulator epithelial splicing regulatory protein 1 to promote epithelial-mesenchymal transition. J Biol Chem.

[13] Brown RL, Reinke LM, Damerow MS, Perez D, Chodosh LA, Yang J (2011). CD44 splice isoform switching in human and mouse epithelium is essential for epithelial-mesenchymal transition and breast cancer progression. J Clin Invest.

[14] Morel AP, Lievre M, Thomas C, Hinkal G, Ansieau S, Puisieux A (2008). Generation of breast cancer stem cells through epithelial-mesenchymal transition. PLoS One.

[15] Mani SA, Guo W, Liao MJ, Eaton EN, Ayyanan A, Zhou AY (2008). The epithelial-mesenchymal transition generates cells with properties of stem cells. Cell.

[16] Jadaan DY, Jadaan MM, McCabe JP (2015). Cellular plasticity in prostate cancer bone metastasis. Prostate Cancer.

[17] Liao TT, Yang MH (2017). Revisiting epithelial-mesenchymal transition in cancer metastasis: the connection between epithelial plasticity and stemness. Mol Oncol.

[18] Koeneman KS, Yeung F, Chung LW (1999). Osteomimetic properties of prostate cancer cells: a hypothesis supporting the predilection of prostate cancer metastasis and growth in the bone environment. Prostate.

[19] Coleman RE (2001). Metastatic bone disease: clinical features, pathophysiology and treatment strategies. Cancer Treat Rev.

[20] Mundy GR (2002). Metastasis to bone: causes, consequences and therapeutic opportunities. Nat Rev Cancer.

[21] Rossi M, Battafarano G, D’Agostini M, Del Fattore A (2018). The role of extracellular vesicles in bone metastasis. Int J Mol Sci.

[22] Ara T, Declerck YA (2010). Interleukin-6 in bone metastasis and cancer progression. Eur J Cancer.

[23] Tsukamoto H, Fujieda K, Senju S, Ikeda T, Oshiumi H, Nishimura Y (2018). Immune-suppressive effects of interleukin-6 on t-cell-mediated anti-tumor immunity. Cancer Sci.

[24] Orian-Rousseau V (2010). CD44, a therapeutic target for metastasising tumors. Eur J Cancer.

[25] Tanabe KK, Ellis LM, Saya H (1993). Expression of CD44R1 adhesion molecule in colon carcinomas and metastases. Lancet.

[26] Gunthert U, Hofmann M, Rudy W, Reber S, Zoller M, Haussmann I (1991). A new variant of glycoprotein CD44 confers metastatic potential to rat carcinoma cells. Cell.

[27] Bourguignon LY, Wong G, Earle C, Chen L (2012). Hyaluronan-CD44v3 interaction with Oct4-Sox2-nanog promotes miR-302 expression leading to self-renewal, clonal formation, and cisplatin resistance in cancer stem cells from head and neck squamous cell carcinoma. J Biol Chem.

[28] Lau WM, Teng E, Chong HS, Lopez KA, Tay AY, Salto-Tellez M (2014). CD44v8-10 is a cancer-specific marker for gastric cancer stem cells. Cancer Res.

[29] Kato T, Mizutani K, Kawakami K, Fujita Y, Ehara H, Ito M (2020). CD44v8-10 mRNA contained in serum exosomes as a diagnostic marker for docetaxel resistance in prostate cancer patients. Heliyon.

[30] Yu S, Cai X, Wu C, Wu L, Wang Y, Liu Y (2015). Adhesion glycoprotein CD44 functions as an upstream regulator of a network connecting ERK, AKT and Hippo-YAP pathways in cancer progression. Oncotarget.

[31] Schmitt M, Metzger M, Gradl D, Davidson G, Orian-Rousseau V (2015). CD44 functions in Wnt signaling by regulating LRP6 localization and activation. Cell Death Differ.

[32] Chan SW, Lim CJ, Guo K, Ng CP, Lee I, Hunziker W (2008). A role for TAZ in migration, invasion, and tumorigenesis of breast cancer cells. Cancer Res.

[33] Deel MD, Li JJ, Crose LE, Linardic CM (2015). A review: molecular aberrations within hippo signaling in bone and soft-tissue sarcomas. Front Oncol.

[34] Panciera T, Citron A, Di Biagio D, Battilana G, Gandin A, Giulitti S (2020). Reprogramming normal cells into tumour precursors requires ecm stiffness and oncogene-mediated changes of cell mechanical properties. Nat Mater.

[35] Zanconato F, Battilana G, Cordenonsi M, Piccolo S (2016). YAP/TAZ as therapeutic targets in cancer. Curr Opin Pharmacol.

[36] Moroishi T, Hansen CG, Guan KL (2015). The emerging roles of YAP and TAZ in cancer. Nat Rev Cancer.

[37] Zeng Y, Wodzenski D, Gao D, Shiraishi T, Terada N, Li Y (2013). Stress-response protein RBM3 attenuates the stem-like properties of prostate cancer cells by interfering with CD44 variant splicing. Cancer Res.

[38] Warzecha CC, Jiang P, Amirikian K, Dittmar KA, Lu H, Shen S (2010). An ESRP-regulated splicing programme is abrogated during the epithelial-mesenchymal transition. EMBO J.

[39] Oshima RG, Baribault H, Caulin C (1996). Oncogenic regulation and function of keratins 8 and 18. Cancer Metastasis Rev.

[40] Kumar B, Koul S, Khandrika L, Meacham RB, Koul HK (2008). Oxidative stress is inherent in prostate cancer cells and is required for aggressive phenotype. Cancer Res.

[41] Cancer Genome Atlas Research Network (2015). The molecular taxonomy of primary prostate cancer. Cell.

[42] Tang Z, Kang B, Li C, Chen T, Zhang Z (2019). GEPIA2: an enhanced web server for large-scale expression profiling and interactive analysis. Nucleic Acids Res.

[43] Bonnomet A, Syne L, Brysse A, Feyereisen E, Thompson EW, Noel A (2012). A dynamic in vivo model of epithelial-to-mesenchymal transitions in circulating tumor cells and metastases of breast cancer. Oncogene.

[44] Akech J, Wixted JJ, Bedard K, van der Deen M, Hussain S, Guise TA (2010). Runx2 association with progression of prostate cancer in patients: mechanisms mediating bone osteolysis and osteoblastic metastatic lesions. Oncogene.

[45] Dougall WC (2012). Molecular pathways: osteoclast-dependent and osteoclast-independent roles of the RANKL/RANK/OPG pathway in tumorigenesis and metastasis. Clin Cancer Res.

[46] Kovacs B, Vajda E, Nagy EE (2019). Regulatory effects and interactions of the Wnt and OPG-RANKL-RANK signaling at the bone-cartilage interface in osteoarthritis. Int J Mol Sci.

[47] Hall CL, Bafico A, Dai J, Aaronson SA, Keller ET (2005). Prostate cancer cells promote osteoblastic bone metastases through Wnts. Cancer Res.

[48] Azzolin L, Zanconato F, Bresolin S, Forcato M, Basso G, Bicciato S (2012). Role of TAZ as mediator of Wnt signaling. Cell.

[49] Udagawa N, Takahashi N, Katagiri T, Tamura T, Wada S, Findlay DM (1995). Interleukin (IL)-6 induction of osteoclast differentiation depends on IL-6 receptors expressed on osteoblastic cells but not on osteoclast progenitors. J Exp Med.

[50] Butte MJ, Keir ME, Phamduy TB, Sharpe AH, Freeman GJ (2007). Programmed death-1 ligand 1 interacts specifically with the B7-1 costimulatory molecule to inhibit T cell responses. Immunity.

[51] Shen MM, Abate-Shen C (2010). Molecular genetics of prostate cancer: new prospects for old challenges. Genes Dev.

[52] Bae KM, Parker NN, Dai Y, Vieweg J, Siemann DW (2011). E-cadherin plasticity in prostate cancer stem cell invasion. Am J Cancer Res.

[53] Fontana F, Raimondi M, Marzagalli M, Sommariva M, Limonta P, Gagliano N (2019). Epithelial-to-mesenchymal transition markers and CD44 isoforms are differently expressed in 2D and 3D cell cultures of prostate cancer cells. Cells.

[54] Yilmaz M, Christofori G (2010). Mechanisms of motility in metastasizing cells. Mol Cancer Res.

[55] Gagliano N, Sforza C, Sommariva M, Menon A, Conte V, Sartori P (2017). 3D-spheroids: what can they tell us about pancreatic ductal adenocarcinoma cell phenotype?. Exp Cell Res.

[56] Pastushenko I, Brisebarre A, Sifrim A, Fioramonti M, Revenco T, Boumahdi S (2018). Identification of the tumour transition states occurring during EMT. Nature.

[57] Gao Y, Bado I, Wang H, Zhang W, Rosen JM, Zhang XH (2019). Metastasis organotropism: redefining the congenial soil. Dev Cell.

[58] Khandrika L, Kumar B, Koul S, Maroni P, Koul HK (2009). Oxidative stress in prostate cancer. Cancer Lett.

[59] Juan-Rivera MC, Martinez-Ferrer M (2018). Integrin inhibitors in prostate cancer. Cancers (Basel).

[60] Lee YC, Jin JK, Cheng CJ, Huang CF, Song JH, Huang M (2013). Targeting constitutively activated beta1 integrins inhibits prostate cancer metastasis. Mol Cancer Res.

[61] Katagiri YU, Sleeman J, Fujii H, Herrlich P, Hotta H, Tanaka K (1999). CD44 variants but not CD44s cooperate with beta1-containing integrins to permit cells to bind to osteopontin independently of arginine-glycine-aspartic acid, thereby stimulating cell motility and chemotaxis. Cancer Res.

[62] Collins AT, Habib FK, Maitland NJ, Neal DE (2001). Identification and isolation of human prostate epithelial stem cells based on alpha(2)beta(1)-integrin expression. J Cell Sci.

[63] Hiraga T, Ito S, Nakamura H (2013). Cancer stem-like cell marker CD44 promotes bone metastases by enhancing tumorigenicity, cell motility, and hyaluronan production. Cancer Res.

[64] Hiraga T, Nakamura H (2016). Comparable roles of CD44v8-10 and CD44s in the development of bone metastases in a mouse model. Oncol Lett.

[65] Chua CW, Chiu YT, Yuen HF, Chan KW, Man K, Wang X (2009). Suppression of androgen-independent prostate cancer cell aggressiveness by FTY720: validating Runx2 as a potential antimetastatic drug screening platform. Clin Cancer Res.

[66] Gupta A, Cao W, Chellaiah MA (2012). Integrin alphavbeta3 and CD44 pathways in metastatic prostate cancer cells support osteoclastogenesis via a Runx2/Smad 5/receptor activator of NF-kappaB ligand signaling axis. Mol Cancer.

[67] Senbanjo LT, AlJohani H, AlQranei M, Majumdar S, Ma T, Chellaiah MA (2020). Identification of sequence-specific interactions of the CD44-intracellular domain with RUNX2 in the transcription of matrix metalloprotease-9 in human prostate cancer cells. Cancer Drug Resist.

[68] Senbanjo LT, AlJohani H, Majumdar S, Chellaiah MA (2019). Characterization of CD44 intracellular domain interaction with RUNX2 in PC3 human prostate cancer cells. Cell Commun Signal.

[69] Ouhtit A, Rizeq B, Saleh HA, Rahman MM, Zayed H (2018). Novel CD44-downstream signaling pathways mediating breast tumor invasion. Int J Biol Sci.

[70] Gori F, Superti-Furga A, Baron R (2016). Bone formation and the Wnt signaling pathway. N Engl J Med.

[71] Xiong J, Almeida M, O’Brien CA (2018). The YAP/TAZ transcriptional co-activators have opposing effects at different stages of osteoblast differentiation. Bone.

[72] Byun MR, Hwang JH, Kim AR, Kim KM, Hwang ES, Yaffe MB (2014). Canonical Wnt signalling activates TAZ through PP1A during osteogenic differentiation. Cell Death Differ.

[73] Liao J, Liu PP, Hou G, Shao J, Yang J, Liu K (2017). Regulation of stem-like cancer cells by glutamine through beta-catenin pathway mediated by redox signaling. Mol Cancer.

[74] Knerr K, Ackermann K, Neidhart T, Pyerin W (2004). Bone metastasis: osteoblasts affect growth and adhesion regulons in prostate tumor cells and provoke osteomimicry. Int J Cancer.

[75] Axmann R, Bohm C, Kronke G, Zwerina J, Smolen J, Schett G (2009). Inhibition of interleukin-6 receptor directly blocks osteoclast formation in vitro and in vivo. Arthritis Rheum.

[76] Tsukamoto H, Fujieda K, Miyashita A, Fukushima S, Ikeda T, Kubo Y (2018). Combined blockade of IL6 and PD-1/PD-L1 signaling abrogates mutual regulation of their immunosuppressive effects in the tumor microenvironment. Cancer Res.

