# Supplementary material for: CD44v8-10 is a marker for malignant traits and a potential driver of bone metastasis in a subpopulation of prostate cancer cells
Source: Cancer Biol Med. 2021 Aug 15;18(3):788–807. doi: 10.20892/j.issn.2095-3941.2020.0495 (PMC8330537; doi:10.20892/j.issn.2095-3941.2020.0495)
Supplement: Supplementary file 1 [file cbm-18-788-s001.pdf]

Supplementary materials

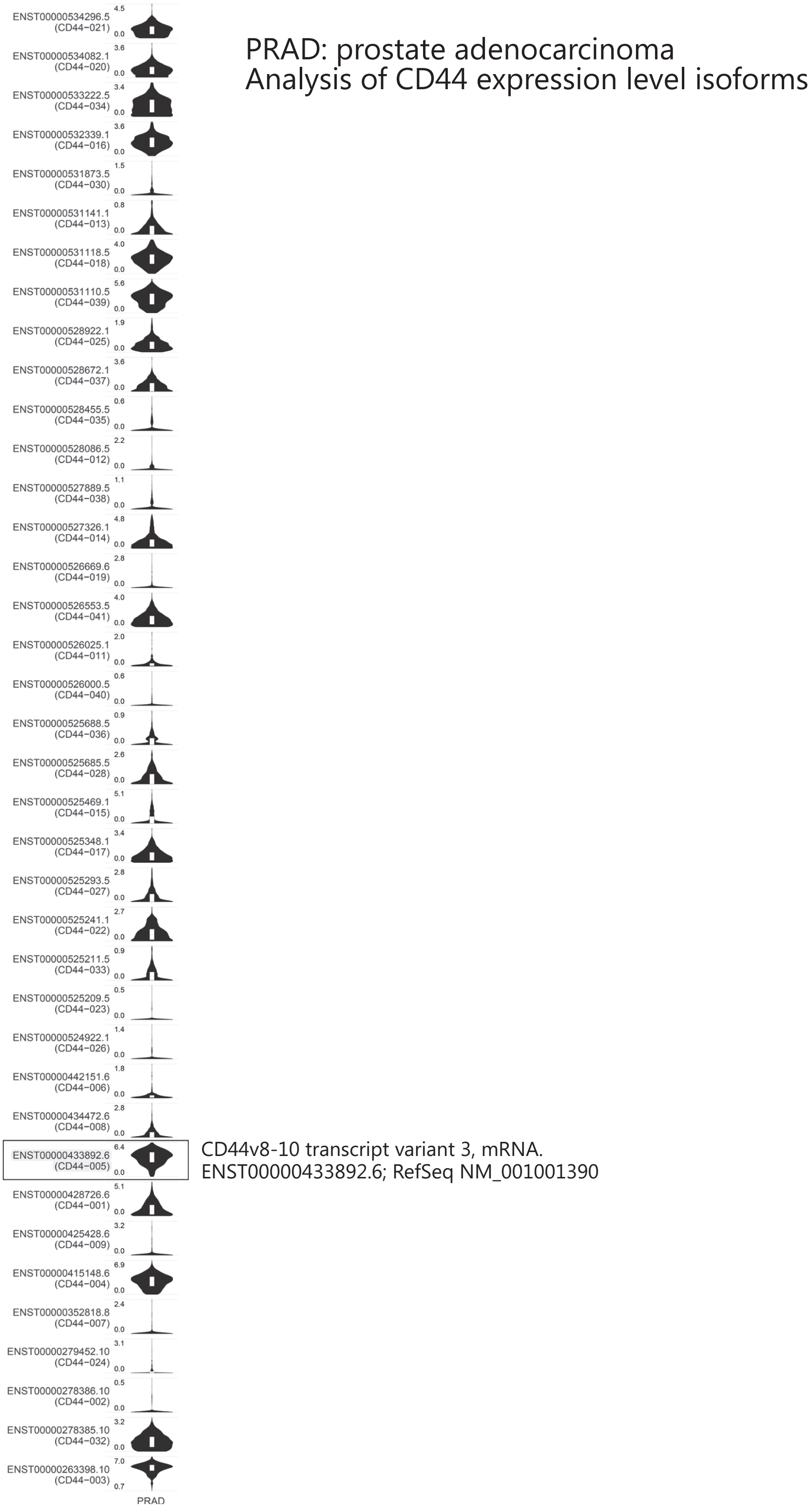

Figure S1 Inhibition of CD44v8-10 expression by specific siRNA treatment.

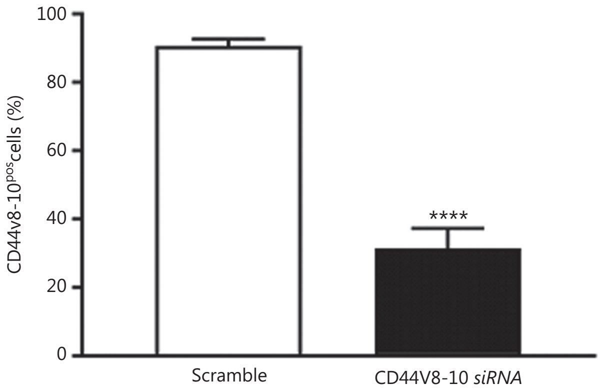

Figure S2 Violin plots showing the expression of CD44 isoforms in PRAD TGCA datasets (*n* = 492). The box-plots (white) represent the mRNA expression. The violin plot outlines (black) illustrate kernel probability density. The CD44v8-10 isoform is indicated in the figure. Scale of the graphs: log10.

Table S1 List of primers used for gene expression analysis

Target gene	Forward primer	Reverse primer
PPARγ	5′-TCAAACGAGAGTCAGCCTTTAACG-3′	5′-AGTGGGAGTGGTCTTCCATTACG-3′
RUNX2	5′-CCCGTGGCCTTCAAGGT-3′	5′-CGTTACCCGCCATGACAGTA-3′
RUNX2	5′-TTACTTACACCCCGCCAGTC-3′	5′-TATGGAGTGCTGCTGGTCTG-3′
β-actin	5′-GAGGCCCAGAGCAAGAGAG-3′	5′-AGGTGTGGTGCCAGATTTTC-3′
H3	5′-GTGAAGAAACCTCATCGTTACAGGCCTGGT-3′	5′-CTGCAAAGCACCAATAGCTGCACTCTGGAA-3′
ANKRD1	5′-CTGTGAGGCTGAACCGCTAT-3′	5′- CTGTGAGGCTGAACCGCTAT-3′
Cyr61	5′-AGCCTCGCATCCTATACAACC-3′	5′-TTCTTTCACAA GGCGGCACTC-3′
TAZ (WWTR1)	5′-CCATCACTAATAATAGCTCAGATCG-3′	5′-GTGATTACAGCCAGG TTAGAAAG-3′
Col1A1	5′- GATTCCCTGGACCTAAAGGTGC-3′	5′- AGCCTCTCCATCTTTGCCAGCA-3′
Col1A2	5′- CCTGGTGCTAAAGGAGAAAGAGG-3′	5′- ATCACCACGACTTCCAGCAGGA-3′
MPP9	5′-GCCACTACTGTGCCTTTGAGTC-3′	5′-CCCTCAGAGAATCGCCAGTACT-3′
MPP13	5′-AAGGAGCATGGCGACTTCT-3′	5′-TGGCCCAGGAGGAAAAGC-3′
IL-6	5′-ATGAACTCCTTCTCCACAAGC-3′	5′-GTTTTCTGCCAGTGCCTCTTTG-3′
